# It takes two: Aberrant repair and low-grade inflammation characterize bronchiolitis obliterans syndrome after lung transplantation in serum proteomic analysis

**DOI:** 10.1016/j.jhlto.2025.100303

**Published:** 2025-05-29

**Authors:** Eline A. van der Ploeg, Alen Faiz, Greta J. Teitsma, Alejandro Sánchez Brotons, Natalia Govorukhina, Jannie M.B. Sand, Diana J. Leeming, Barbro N. Melgert, Peter Horvatovich, Janette K. Burgess, C. Tji Gan

**Affiliations:** a Department of Pulmonary Medicine, University of Groningen, University Medical Center Groningen, Groningen, the Netherlands; b Respiratory Bioinformatics and Molecular Biology (RBMB), School of Life Sciences, University of Technology Sydney, Sydney, Australia; c Groningen Research Institute for Asthma and COPD (GRIAC), University of Groningen, University Medical Center Groningen, Groningen, the Netherlands; d Department of Pathology and Medical Biology, University of Groningen, University Medical Center Groningen, Groningen, the Netherlands; e Department of Analytical Biochemistry, Groningen Research Institute of Pharmacy, University of Groningen, Groningen, the Netherlands; f Hepatic and Pulmonary Research, Nordic Bioscience, Herlev, Denmark; g Department of Molecular Pharmacology, Groningen Research Institute of Pharmacy, University of Groningen, Groningen, the Netherlands

**Keywords:** bronchiolitis obliterans syndrome, chronic lung allograft dysfunction, lung transplantation, fibrosis, inflammation

## Abstract

**Background:**

The obstructive phenotype of chronic lung allograft dysfunction, bronchiolitis obliterans syndrome (BOS), is diagnosed after lung transplantation (LTx) when irreversible airway obstruction is already present. This study aimed to investigate biomarkers indicative of aberrant repair resulting in a fibrotic response and inflammation signals in the serum of patients with BOS.

**Methods:**

LTx patients transplanted at the University Medical Center Groningen between 2004 and 2017 were screened. Nineteen patients with BOS were selected and matched with 19 patients with non-BOS. Only patients for whom lung function and longitudinal serum samples post-LTx were available were included. Enzyme-linked immunosorbent assays were performed for neoepitopes of collagen types I, III, and VI and osteoprotegerin (OPG) in serum. Additionally, serum samples were analyzed by label-free liquid chromatography with tandem mass spectrometry proteomics analysis.

**Results:**

Collagen neoepitopes did not differ significantly between patients with BOS and non-BOS at any timepoint. OPG was significantly higher in non-BOS compared to BOS 6 months before BOS onset (*p* < 0.04). In proteomics analysis, proteins indicating cell repair and proliferation, namely human type II keratin-6 and centromere protein F (both FDR < 0.1), were significantly lower 3 months before BOS onset in patients with BOS compared to patients with non-BOS. C-reactive protein (FDR < 0.05) and SERPINA3 (FDR < 0.05), among others, were higher in end-stage patients with BOS compared to patients with non-BOS.

**Conclusions:**

Differences in the expression of proteins that reflect the complex interplay between aberrant repair and inflammation in BOS were identified. These proteins should be investigated and validated in larger cohorts and may aid in expanding knowledge about the development of BOS.

## Background

Lung transplantation (LTx) is a life-saving treatment for several lung diseases, such as chronic obstructive pulmonary disease and idiopathic pulmonary fibrosis (IPF).^1^ Annually, 3,800 LTx are performed worldwide^2^; however, long-term survival is restricted mainly by the development of chronic lung allograft dysfunction (CLAD). CLAD affects half of all patients 5 years after LTx, causing major morbidity and mortality.^3^, ^4^

Two main forms of CLAD can be identified: restrictive allograft syndrome (RAS) and bronchiolitis obliterans syndrome (BOS).^5^ RAS is characterized by restrictive pulmonary function reflected by rapid decline of forced expiratory volume in 1 second (FEV1) and total lung capacity as well as persistent pulmonary opacities on computed tomography. In BOS, the most common form of CLAD, obstructive pulmonary function is the hallmark for diagnosis in absence of other factors such as infection. Patients develop a persistent decline in FEV1 and FEV1/forced vital capacity ratio representing irreversible damage.^5^ BOS is pathologically characterized by obliterative bronchiolitis that causes progressive obliteration of small airway lumina in the donor lung.^5^ The patchy occurrence of the fibrotic lesions complicates early pathological diagnosis.^3^

The pathogenesis of BOS remains only partially understood. Allo-immune dependent as well as independent factors, such as infection and gastro-esophageal reflux, are known to contribute to the complex pathogenesis.^6^, ^7^ Repeated damage to the airway induces recruitment of neutrophils as well as (myo)fibroblasts, among others. Aberrant repair characterized by epithelial to mesenchymal transition, tissue remodeling, and excess production of extracellular matrix, including accumulation of collagens, collectively progress fibrotic obliteration of the airway.^8^, ^9^ Changes in extracellular matrix composition can be seen in biopsies from LTx patients before BOS onset compared to controls, highlighting the importance of the extracellular matrix in BOS.^10^ Earlier research in IPF showed increased levels of N-terminal propeptide of type III collagen (PRO-C3), N-terminal propeptide of type VI collagen (PRO-C6), a fragment of type I collagen released by MMP (C1M), and a fragment of type VIα1 collagen released by MMP-2 (C6M) in serum, all markers for collagen types I, III and VI turnover, that related to progression of disease.^11^, ^12^, ^13^, ^14^, ^15^ Also, osteoprotegerin (OPG), known for its role in matrix turnover and instigating fibrogenesis through upregulation of TGF-β1,^16^ was described to be increased in human and mouse fibrotic lung tissue and to associate with progressive fibrotic disease.^17^

Since excess production of extracellular matrix, particularly accumulation of collagens, is a hallmark of BOS, we hypothesized that these blood biomarkers could also reflect disease in BOS, possibly before onset of lung function decline, and with progression of disease. We also explored hypothesis-free protein profiling through mass spectrometry of serum of LTx patients with BOS and non-BOS could provide novel insights into the underlying fibrotic molecular and cellular processes driving BOS before BOS onset, as well as in progression of disease. Therefore, we performed label-free proteomics on serum of patients with BOS and matched non-BOS patients including samples before and after BOS onset. Within this dataset, all proteins were analyzed, with no protein set of interest being chosen beforehand (hypothesis-free). As such, we did not focus on proteins involved in fibrotic or repair pathways in the serum proteomics analysis only, but wanted to identify potential novel pathways underlying BOS. We did hypothesize, however, that we would find a signal pointing toward aberrant repair and fibrotic response in BOS due to the underlying pathogenesis of BOS. To our knowledge, label-free serum proteomics and serum analysis of collagen turnover have not been performed in BOS before.

## Methods

### Study design and patient selection

**Figure 1:**
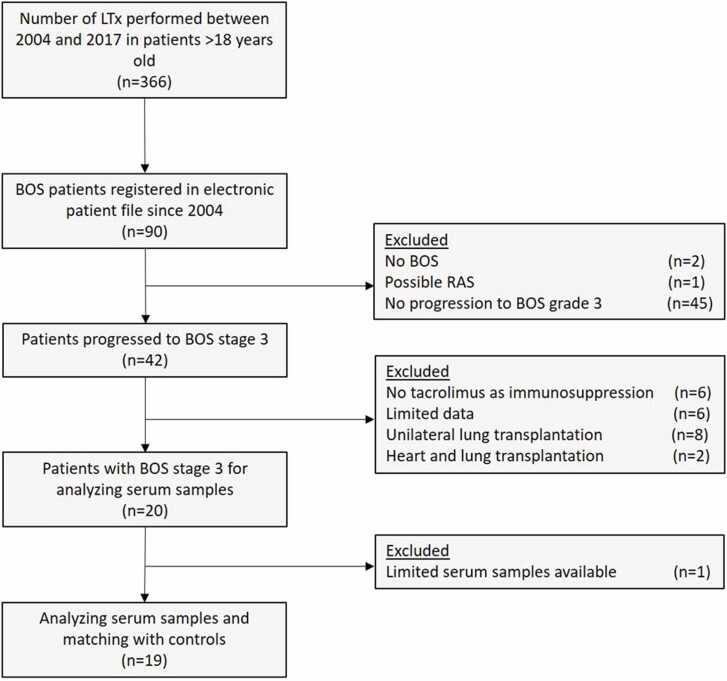
Flowchart indicating patient selection for BOS patients matched to non-BOS patients. Abbreviations: LTx: lung transplantation; BOS: bronchiolitis obliterans syndrome; LTx, lung transplantation; n, number; RAS, restrictive allograft syndrome.

For this retrospective study, LTx patients of 18 years and older who underwent bilateral transplantation between 2004 and 2017 in the University Medical Center Groningen, the Netherlands, were screened for eligibility. We started screening from 2004 onward because of the introduction of tacrolimus. Nineteen patients who developed BOS and who all progressed to BOS stage 3 ascertained on International Society of Heart and Lung Transplantation (ISHLT) Guidelines^18^ were selected based on availability of at least 3 historically collected longitudinal serum samples (Figure 1). In our center, historically, serum samples were collected from patients during follow-up after LTx at outpatient clinic visits or when patients were admitted. Patients with mixed CLAD phenotypes were excluded. Patients with BOS were matched with 19 non-BOS LTx patients. Patients were matched for sex, age at LTx, disease necessitating LTx, type of immunosuppression used, date of LTx, and total storage time of samples. At the timepoints of data and serum collection, other causes of lung function decline were excluded according to ISHLT guidelines.^18^ All patients received standardized immunosuppression, pulmonary function follow-up, and clinical management according to the local LTx protocol. Patients provided written informed consent for use of material. The study was approved by the medical ethics committee of the University Medical Centre Groningen (METc 2021/610, research register number: 202000737).

### Sample collection

At the timepoints of data collection, other causes of lung function decline were excluded. Timepoints for serum sample collection of patients were 12, 6, and 3 months before BOS onset (BOS stage 1), at BOS stage 1, at BOS stage 2, and BOS stage 3. BOS grading was defined according to the ISHLT guideline.^18^ For non-BOS patients, dates of the serum sample collections were matched in relation to time since LTx date compared to the patients with BOS.

**Figure 2:**
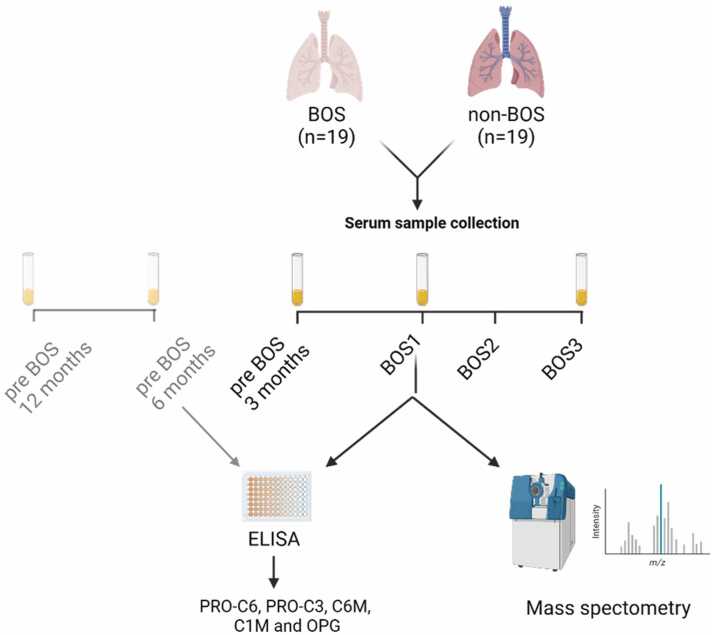
Flow diagram of methods used for analysis of serum samples of patients with BOS and non-BOS patients. For OPG and collagen fragment analysis, samples were collected at 12, 6, and 3 months before onset of BOS, at BOS stages 1, and 3 (BOS *n* = 19 and non-BOS *n* = 19). Samples from patients with BOS and non-BOS were collected at 3 months before BOS onset, BOS stages 1, and 3 for analyses with mass spectrometry (BOS *n* = 18, non-BOS *n* = 16). BOS, bronchiolitis obliterans syndrome; BOS1, BOS stage 1; BOS2, BOS stage 2; BOS3, BOS stage 3; C1M, type I collagen degradation; C6M, type VI collagen degradation; OPG, osteoprotegerin; PRO-C3, type III collagen formation; PRO-C6, type VI collagen formation. Figure composed with Biorender.

Serum samples from 19 patients with BOS and 19 patients with non-BOS were analyzed by multiple ELISAs for collagen neoepitope testing for all timepoints available. Serum samples of 18 patients with BOS and 16 patients with non-BOS were analyzed by label-free proteomics analysis (Figure 2). Proteomics was performed on samples at timepoints minus 3 months before BOS onset, BOS stage 1, and BOS stage 3 samples in patients with BOS and non-BOS patients. Missing samples were not replaced (Tables S1 and S2).

### OPG and collagen neoepitopes

OPG was measured in serum using a human OPG DuoSet ELISA kit (R&D Systems, Minneapolis, MN) according to the instructions provided by the manufacturer. Collagen neoepitopes C1M, C6M, PRO-C3, and PRO-C6 were measured in serum using neoepitope-specific competitive ELISAs developed and validated by Nordic Bioscience (Herlev, Denmark) according to previously published protocols.

C1M and C6M quantify type I and VI collagen degradation fragments, respectively, generated by matrix metalloproteinases and were measured using the nordicC1M and nordicC6M assays.^19^, ^20^ PRO-C3 and PRO-C6 quantify type III and VI collagen formation, respectively, and were measured using the nordicPRO-C3 and nordicPRO-C6 assays.^21^, ^22^

### Proteomics

**Figure 3:**
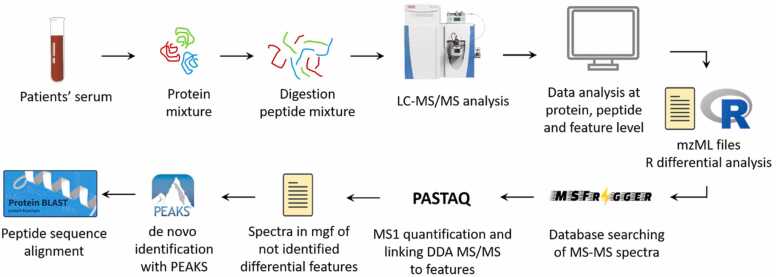
Flow diagram for proteomics analysis on serum samples of patients with BOS and non-BOS patients. Patients’ serum was depleted and analyzed by LC-MS/MS analysis. Data retrieved were processed with R, MSfragger, and PASTAQ, after which de novo identification for proteins with PEAKS was performed. DDA, data-dependent analysis; LC-MS/MS, liquid chromatography with tandem mass spectrometry; mgf, mascot generic format; MS-MS, tandem mass spectrometry.

The proteomic analysis was performed on serum samples after depletion of the most abundant proteins, such as albumin. Proteins were digested with the use of trypsin. Using liquid chromatography with tandem mass spectrometry (LC-MS/MS) analysis, samples were analyzed, and data retrieved. Peptide and protein identification was performed using a false discovery rate of 1% at peptide-spectrum matching, peptide and protein levels. Human proteome from Swissprot was used for identification of proteome, after which quantitative processing was performed with PASTAQ^23^ (Figure 3, for a detailed description of proteomics methodology see Supplement 1).

### C-reactive protein collection

C-reactive protein (CRP) is routinely measured using turbidimetry during follow-up visits in the University Medical Center Groningen. For validation of proteomics results, CRP data were retrospectively collected from electronic patients’ files.

### Statistical analyses

Statistical analyses were performed using RStudio 2021.09.1 Build 372 and IBM SPSS version 28.0. Differential expression analyses were performed using the Limma R package across peptide, protein, and feature data sets. All proteomic analyses were corrected for sex, age, and alpha-1 antitrypsin deficiency, to check for associations between these variables, BOS, and protein levels. False discovery rate for peptides was set at 0.05. For protein and feature level, the false discovery rate was set at 0.1 to allow for identification of new proteins. Data for proteomics analysis were log2 transformed for normalization, and a median scale normalization was conducted. Proteins that were not expressed in 25% of the total samples were filtered out. Continuous data are represented as mean ± standard deviation or median [interquartile range], depending on normality. Differences in patient characteristics between patients with BOS and non-BOS were analyzed by Mann-Whitney U test for continuous not normally distributed data and chi-square test for categorical data. Independent samples *t*-test was used to analyze continuous normally distributed data. ELISA results between groups were analyzed at each timepoint with Mann-Whitney U test for matched data and Wilcoxon-signed rank test for paired data within groups. A *p*-value <0.05 was considered significant.

## Results

### Patient characteristics

**Figure 4:**
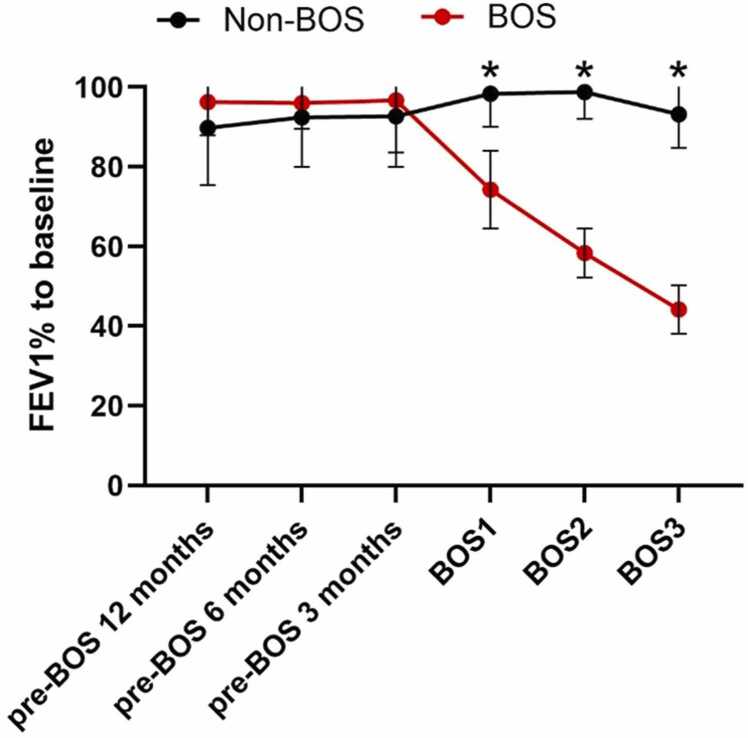
Forced expiratory volume in 1 second in percentage compared to baseline measurements for patients with BOS and non-BOS patients. Baseline measurements were defined as the average of the best 2 measurements post lung transplantation with at least 3 weeks separation. At BOS stages 1, 2, and 3, FEV1% compared to baseline was significantly different between patients with BOS and non-BOS when analyzed with unpaired *t*-test (BOS stage 1: 74.3 ± 9.8 vs 95.5 ± 12.7, *p* < 0.001; BOS stage 2: 58.4 ± 6.2 vs 98.8 ± 6.8, *p* < 0.001; BOS stage 3: 44.2 ± 5.9 vs 93.2 ± 8.4, *p* < 0.001). All error bars reflect mean ± standard deviation. BOS, bronchiolitis obliterans syndrome; BOS1, BOS stage 1; BOS2, BOS stage 2; BOS3, BOS stage 3. *p-value <0.05.

The median age at LTx was 55 years, with chronic obstructive pulmonary disease being the most common reason for LTx, followed by alpha-1 antitrypsin deficiency (Table 1). Patients developed BOS 2.8 [1.9-5.8] years after LTx, underlying disease pre-LTx did not affect time to BOS onset. There was no significant difference in time between LTx and serum samples collected between patients with BOS and non-BOS for all timepoints (*p* > 0.2, Table S3). FEV1 and forced vital capacity in liters, as well as in percentage predicted, differed significantly from the onset of BOS diagnosis in patients with BOS compared to patients with non-BOS, supporting diagnosis (Figure S1). FEV1 as percentage from baseline post-LTx did not differ between patients with BOS and non-BOS at timepoints before BOS onset; however, a steady decline was observed from BOS onset in BOS group compared to patients with non-BOS reflecting BOS diagnosis (Figure 4).

**Table 1:** Patient Characteristics of Patients With BOS and Non-BOS patients

	BOS (*n* = 19)	Non-BOS (*n* = 19)	*p*-value
Patient sex, female (%)	12 (63.2%)	12(63.2%)	1.0
Age at LTx (years)	55 [44-66]	55 [43-66]	0.6
Underlying disease			0.7
COPD	9^a^	11^a^	
CF	2	2	
AATD	5	3	
Scleroderma	1	1	
Other	2^b^	2^b^	
Donor sex, female (%)	12 (63.2%)	11 (57.9%)	1.0
Immunosuppression after LTx			1.0
Tacrolimus/azathioprine/prednisolone	5 (26.3%)	5 (26.3%)	
Tacrolimus/MMF/prednisolone	14 (73.7%)	14 (73.7%)	
Patients with documented viral infection after LTx	10 (52.6%)	3 (15.8%)	0.08
Time to development of BOS (years)	2.8 [1.9-5.8]	NA	

Abbreviations: AATD, alpha-1 antitrypsin deficiency; BOS, bronchiolitis obliterans syndrome; CF, cystic fibrosis; COPD, chronic obstructive pulmonary disease; LTx, lung transplantation; MMF, mycophenolic acid; n, number; y, years.

a Patients with BOS with alpha-1 antitrypsin deficiency were matched to non-BOS COPD controls.

b One patient with BOS with histiocytosis X matched with a patient with non-BOS with pulmonary fibrosis. One patient with BOS with pulmonary fibrosis after infection matched with a patient with non-BOS with selective IgG2 deficiency and bronchiectasis.

### Fibrosis markers osteoprotegerin and collagen neoepitopes are not associated with BOS

**Figure 5:**
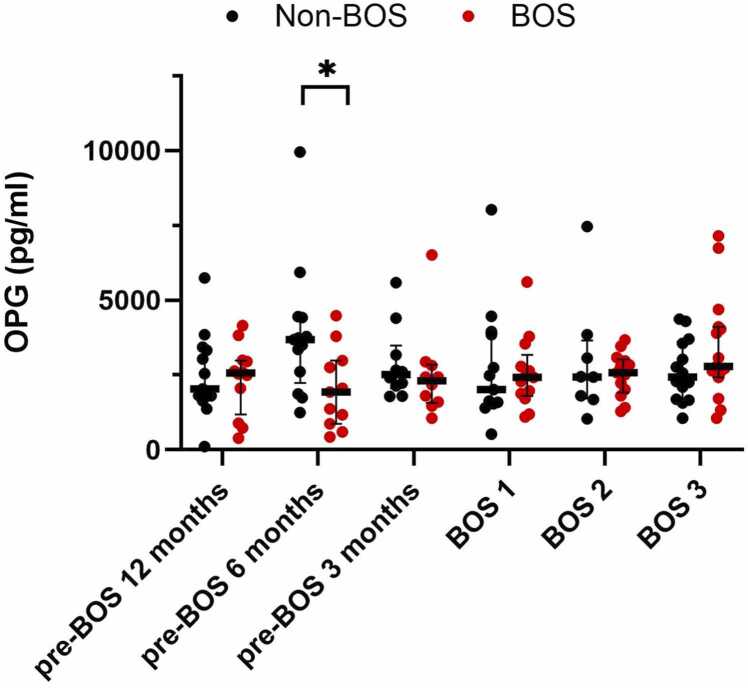
Osteoprotegerin (OPG) serum levels measured with ELISA in patients with BOS and non-BOS patients (BOS *n* = 19, non-BOS *n* = 19). Mann-Whitney U was used to compare serum levels between groups at different timepoints. All error bars reflect median [interquartile range]. Longitudinal differences within BOS and non-BOS groups were analyzed with Wilcoxon-signed rank test. BOS, bronchiolitis obliterans syndrome; BOS1, BOS stage 1; BOS2, BOS stage 2; BOS3, BOS stage 3. *p-value <0.05.

**Figure 6:**
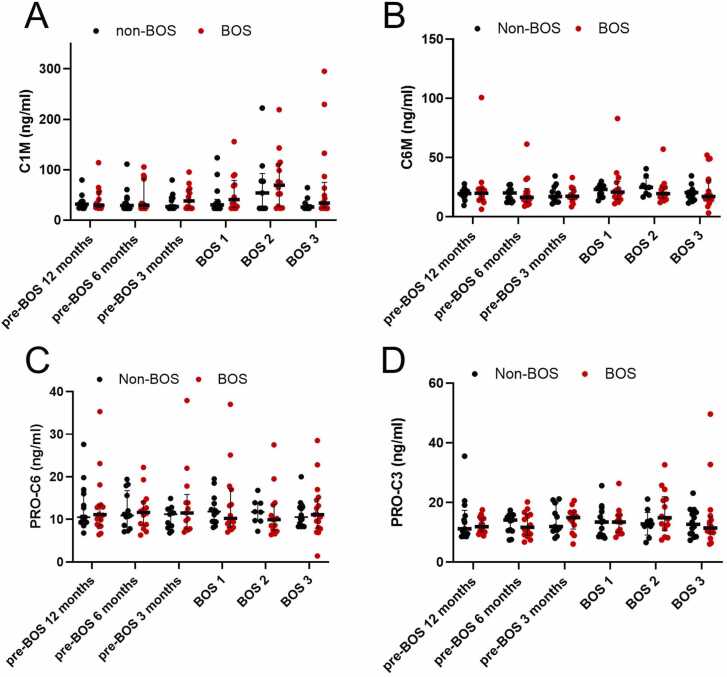
Serum levels of collagen fragments C1M, C6M, PRO-C3, and PRO-C6 at different timepoints for patients with BOS and non-BOS patients (BOS *n* = 19, non-BOS *n* = 19). Differences between patients with BOS and non-BOS were analyzed with Mann-Whitney U test at each timepoint. Longitudinal differences within BOS and non-BOS groups were analyzed with Wilcoxon-signed rank test. All error bars reflect median with interquartile range. BOS, bronchiolitis obliterans syndrome; BOS1, BOS stage 1; BOS2, BOS stage 2; BOS3, BOS stage 3; C1M, type I collagen degradation; C6M, type VI collagen degradation; PRO-C3, type III collagen formation; PRO-C6, type VI collagen formation.

OPG serum levels were significantly lower in patients developing BOS 6 months before BOS onset compared to patients with non-BOS (1,932 vs 3,685 pg/ml, *p* = 0.04). However, this analysis was not significant after exclusion of the outlier datapoint from 1 patient with non-BOS. OPG levels were not different at other timepoints (Figure 5). For the collagen neoepitopes, neither C1M, C6M, PRO-C6, or PRO-C3 serum levels differed in patients with BOS compared to patients with non-BOS (*p* > 0.05 across all timepoints; Figure 6). C1M levels appeared to be higher in patients with BOS compared to patients with non-BOS at all timepoints; however, ROC analysis did not show predictive value for any of the timepoints for C1M, neither were there significant changes found across BOS stages (data not shown).

### Proteomics analysis

To identify potentially new fibrotic and inflammatory markers for development of BOS, we used a hypothesis-free approach for serum protein profiling. Through LC-MS/MS, serum samples from patients with BOS and non-BOS were analyzed 3 months before BOS onset, at BOS stage 1, and BOS stage 3, after depletion of most abundant proteins such as albumin.

#### NCHL1, K2C6A, and CENPF expression was decreased, while FANCE expression was increased in patients with BOS compared to patients with non-BOS 3 months before BOS

**Figure 7:**
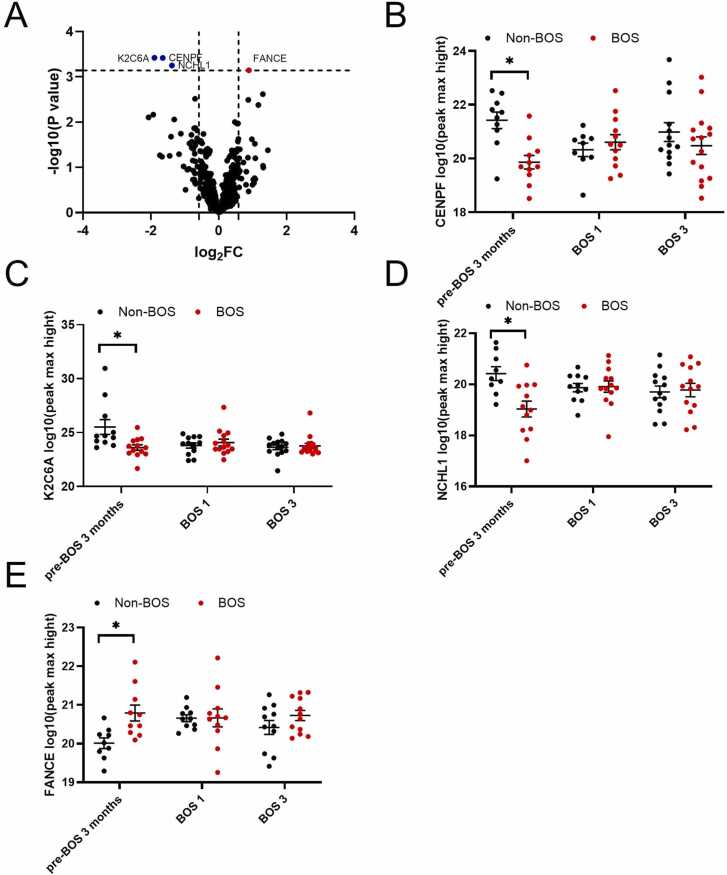
Proteins that were expressed at significantly different protein levels between patients with BOS and non-BOS patients 3 months before onset of BOS (BOS *n* = 18, non-BOS *n* = 16). False discovery rate <0.1 was considered significant. Protein levels were log10 transformed. (A) Overview of significant differentially expressed proteins in proteomic analysis in patients with BOS compared to patients with non-BOS, red indicates overall higher expression, blue indicates lower expression in patients with BOS compared to patients with non-BOS. (B) CENPF expression 3 months pre-BOS, at BOS stage 1, and BOS stage 3 in patients with BOS compared to patients with non-BOS. (C) K2C6A expression pre-BOS, at BOS stage 1, and BOS stage 3 in patients with BOS compared to patients with non-BOS. (D) NCHL1 expression pre-BOS, at BOS stage 1, and BOS stage 3 in patients with BOS compared to patients with non-BOS. (E) FANCE expression 3 months pre-BOS, at BOS stage 1, and BOS stage 3 in patients with BOS compared to patients with non-BOS. All error bars reflect standard error of the mean. BOS, bronchiolitis obliterans syndrome; BOS1, BOS stage 1; BOS3, BOS stage 3; CENPF, centromere protein F; FANCE, Fanconi anemia complementation group E protein; K2C6A, human type II Keratin-6; NCHL1, neural cell adhesion molecule L1-like protein; pre-BOS, 3 months before BOS onset. *p-value <0.05.

Three months before BOS onset, neural cell adhesion molecule L1-like protein (NCHL1), human type II Keratin-6 (K2C6A) and centromere protein F (CENPF) were lower in patients with BOS compared to patients with non-BOS patients(FDR < 0.1, Figure 7, Table 2). Fanconi anemia complementation group E protein (FANCE) expression was higher in patients with BOS compared to patients with non-BOS patients (FDR < 0.1, Figure 7, Table 2). However, these differences did not persist over time for any proteins, indicating a lack of stability of the protein markers.

**Table 2:** Proteins Differentially Expressed at Protein-level Data Three Months Before Onset of Patients With BOS Compared to Non-BOS patients

Peptide	logFC	*p*-value	False discovery rate
CENPF	−1.65	3.83 × 10^−4^	0.0813
K2C6A	−1.90	3.78 × 10^−4^	0.0813
NCHL1	−1.38	5.62 × 10^−5^	0.0813
FANCE	0.887	7.13 × 10^−4^	0.0813

Abbreviations: BOS, bronchiolitis obliterans syndrome; CENPF, centromere protein F; FANCE, Fanconi anemia complementation group E protein; K2C6A, human type II Keratin-6; logFC, log fold change; NCHL1, neural cell adhesion molecule L1-like protein.

False discovery rate <0.1 was considered significant.

### CRP expression is higher at BOS stage 3 in patients with BOS compared to patients with non-BOS

**Figure 8:**
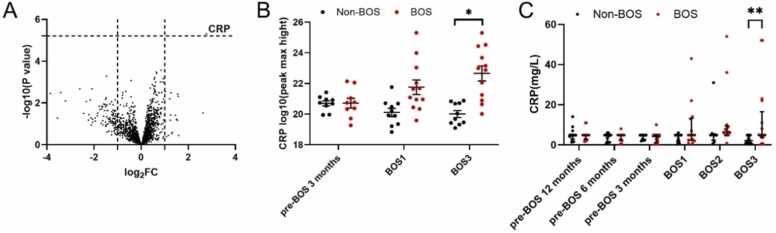
Protein that was expressed significantly different between patients with BOS and non-BOS patients at peptide-level data at BOS stage 3 in proteomic analysis and validated with retrospectively collected patient data. False discovery rate <0.05 was considered significant. (A) An overview of significant differentially expressed protein in proteomic analysis in patients with BOS compared to patients with non-BOS, red indicates overall higher expression in patients with BOS compared to patients with non-BOS. (B) Serum C-reactive levels measured with proteomic analysis in patients with BOS compared to patients with non-BOS 3 months before BOS onset, at BOS stage 1, and BOS stage 3. Peptide levels were log transformed to increase normality. (C) For serum samples collected during regular follow-up, CRP levels reported in mg/liter in patient records were collected and analyzed with Mann-Whitney U (BOS *n* = 17, non-BOS *n* = 14). Error bars reflect standard error of the mean in (B), error bars reflect median with interquartile range in (C). BOS, bronchiolitis obliterans syndrome; BOS1, BOS stage 1; BOS3, BOS stage 3; CRP, C-reactive protein; pre-BOS, 3 months before BOS onset. *p < 0.05; **p < 0.01.

At BOS stage 3, CRP expression was higher in patients with BOS compared to patients with non-BOS at peptide level in LC-MS/MS analysis. Even though a trend toward higher expression of CRP was observed 3 months before BOS onset and at BOS diagnosis, this failed to reach significance in our limited dataset (Figure 8A and B). For validation, CRP serum results from patients with BOS and non-BOS were collected from electronic patient records to compare results to proteomics data. In serum, routinely collected CRP levels at end-stage BOS were significantly higher in patients with BOS compared to patients with non-BOS (Figure 8C, predicted difference 9.5 mg/liter, 95% CI 1.6-17.4, *p* < 0.01). Interestingly, neither leukocyte counts nor neutrophil and lymphocyte counts were significantly different in patients with BOS vs patients with non-BOS at BOS stage 3 (data not shown).

#### SERPINA3, CRP, PZP, ITIH3, and C4BP expression is higher at BOS stage 3 in patients with BOS compared to non-BOS patients

**Figure 9:**
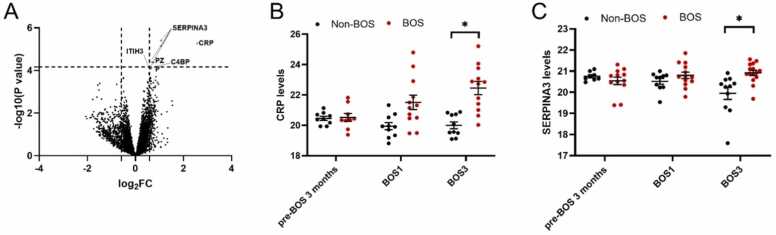
Significant proteins and their expression levels in patients with BOS and non-BOS patients (BOS *n* = 18, non-BOS *n* = 16) at BOS stage 3 at feature level, analyzed from serum proteomics. False discovery rate <0.1 was considered significant. Peptide levels were log transformed to increase normality. (A) Overview of significant differentially expressed peptides in proteomic analysis in patients with BOS compared to patients with non-BOS 3 months before BOS onset. Red indicates overall higher expression in patients with BOS compared to patients with non-BOS. Peptide levels were log transformed to improve normality. (B) Serum C-reactive peptide levels measured with proteomic analysis in patients with BOS compared to patients with non-BOS 3 months before BOS onset, at BOS stage 1, and BOS stage 3. (C) SERPINA3 levels measured with proteomic analysis in patients with BOS compared to patients with non-BOS 3 months before BOS onset, at BOS stage 1, and BOS stage 3. All error bars reflect standard error of the mean. BOS, bronchiolitis obliterans syndrome; BOS1, BOS stage 1; BOS3, BOS stage 3; CRP, C-reactive protein; pre-BOS, 3 months before BOS onset; SERPINA3, SERPIN A Family member 3 . *p < 0.05.

Feature-level analysis did not show significant differences for proteins in patients with BOS compared to patients with non-BOS before BOS onset. However, at BOS stage 3, several features were increased in patients with BOS. These (Figure 9, Table 3) showed that SERPIN Family A member 3 (SERPINA3) and CRP had significantly higher expression in patients with BOS compared to patients with non-BOS (FDR < 0.05). Also, Pregnancy Zone Protein (PZP), Inter-Alpha-Trypsin Inhibitor Heavy Chain 3 (ITIH3), and C4 binding protein (C4BP) expression were significantly higher at end-stage BOS in patients with BOS when compared to patients with non-BOS (FDR < 0.1).

**Table 3:** Protein Expression at Feature Level With Significant Differential Expression at BOS Stage 3 in Patients With BOS Compared to Non-BOS patients.

Peptide	logFC	*p*-value	False discovery rate
SERPINA3 feature 1	1.08	4.02 × 10^−6^	0.0231
SERPINA3 feature 2	1.06	7.21 × 10^−6^	0.0231
CRP	2.58	5.37 × 10^−6^	0.0231
SERPINA3 feature 3	0.76	4.18 × 10^−5^	0.0805
PZP	0.69	4.09 × 10^−5^	0.0805
AACT	0.66	7.13 × 10^−5^	0.0980
ITIH3	0.64	8.24 × 10^−5^	0.0992
C4BP	0.86	6.80 × 10^−5^	0.0980

Abbreviations: AACT, alfa-1-anti-chymotrypsin; BOS, bronchiolitis obliterans syndrome; C4BP, C4 binding protein; CRP, C-reactive protein; ITIH3: Inter-Alpha-Trypsin Inhibitor Heavy Chain 3; logFC, log fold change; PZP, pregnancy zone protein; SERPINA3, SERPIN Family A member 3.

False discovery rate or adjusted p-value <0.1 was considered significant.

## Discussion

In this retrospective cohort study, we found that patients with BOS exhibit both an increase in FANCE expression and decrease in K2C6A expression, suggesting a role for an aberrant repair response, as well as elevated CRP expression, reflecting a low-grade inflammatory response.

Unexpectedly, serum levels of collagen neoepitopes did not differ between patients with BOS and non-BOS patients. We hypothesized that OPG and collagen neoepitopes would be higher in patients with BOS compared to non-BOS patients, as is seen in IPF,^17^, ^24^, ^25^ but could not confirm this hypothesis in our small cohort. Possibly, OPG and collagen fragment levels in serum do not reflect the process of intraluminal fibrosis due to the inability of these markers to be released systemically from localized lesions, the hallmark of BOS. In comparison, the process of alveolar fibrosis in IPF patients is more diffuse and located in closer proximity to the circulation. Possibly, these markers could reflect the pathological process in RAS, reflecting alveolar fibrosis compared to intraluminal obstruction in BOS. Lung tissue analysis with specific staining for these markers in BOS, RAS, and patients with non-BOS may shed a different light on the involvement of remodeling of these specific collagens in CLAD.

NCHL1, K2C6A, and CENPF protein expression were decreased 3 months before BOS onset, while FANCE expression increased. All failed to show persistence during the progression of BOS. Nevertheless, these findings shed new light on potential pathways involved in the early pathogenesis of BOS. NCHL1 is known to have tumor suppressive effects and is associated with prolonged survival in lung cancer.^26^ The role of NCHL1 in healthy lung is unknown. K2C6A also has a yet undefined role in lung cancer cell proliferation and migration.^27^, ^28^ Downregulation of CENPF, a mitotic cell regulator, could result in decreased cell proliferation.^29^ FANCE is one of the 8 proteins that form the core complex needed for monoubiquitination of the FANCD2-FANCI complex. This complex is needed for the intricate process of repairing DNA. Mutations or isoforms of one of the proteins of this core complex lead to increased risk of cancer in certain genetic diseases.^30^ It is not known what an increase in FANCE expression in BOS reflects. In this study, we did not analyze if increased FANCE expression in this cohort is, for example, due to the presence of an alternative isoform. The role of ITIH3, PZP, and C4BP in the process of BOS and in end-stage BOS remains to be elucidated. While ITIH3 is involved in stabilization of the extracellular matrix,^31^ PZP and C4BP both serve as immunosuppressive proteins on, for example, T helper cells and the complement system.^32^, ^33^, ^34^, ^35^ To date, these proteins have not been described in (fibrotic) lung diseases.

We found higher expression of CRP and SERPINA3 in end-stage patients with BOS compared to non-BOS patients. CRP has multiple functions as an immunologic moderator. CRP stimulates phagocytotic cells, activation of the complement system, opsonization, and production of anti-inflammatory cytokines,^36^ while also moderating the migration of fibroblasts.^37^ CRP can increase up to 1,000-fold in acute infection; however, low serum levels are related to low-grade inflammation in rheumatological and cardiovascular disease.^38^, ^39^, ^40^ Interestingly, the levels of CRP in patients with BOS in our cohort remain relatively low and could be attributed to low-grade inflammation. Vos et al showed that increased CRP plasma and bronchoalveolar lavage (BAL) fluid levels 90 days post-LTx were predictive of graft failure 3 years after LTx, underlining its clinical relevance.^41^, ^42^ SERPINA3 is a protease inhibitor that prevents damage from neutrophil elastase released during inflammation.^43^ Recently, SERPINA3 was identified to be higher in severe asthma patients compared to mild asthma patients and healthy controls.^44^ Epithelial to mesenchymal transition, also a well-known pathogenic phenomenon in BOS, was enhanced by SERPINA3 in triple-negative breast cancer and glioblastoma.^45^, ^46^ Future analysis of SERPINA3 lung tissue expression, BAL fluid levels collected prospectively, and validation in a larger cohort in patients with BOS and non-BOS patients’ serum would aid in further elucidating the role of SERPINA3 in BOS.

Even though differences in any of our identified proteins’ expression did not persist with progression of BOS, our results taken together might point toward aberrant repair, fibrogenesis, and modifications in cell life cycles that precede BOS and increased inflammatory status at end-stage BOS. The differentially expressed proteins, such as CRP, FANCE, and CENPF, we identified in this study might refer to this process of increased inflammation, decreased resilience of cells, aberrant cell repair, and increased apoptosis; however, further research is needed to elucidate the role of these proteins in BOS. We also hypothesize that within the complex pathogenesis of BOS, SERPINA3 could be a missing link in the interlude between the intertwined processes of inflammation and fibrosis in BOS due to its known role in epithelial mesenchymal transition and inflammation. Different cohorts, including more patients with more timepoints before and after BOS onset, to investigate the further significance of these proteins in BOS are needed.

This study performed label-free serum proteomics on several timepoints in BOS, yielding new possible markers and pathways to investigate. The historical availability of these sequential serum samples enabled us to perform these analyses on samples before and after onset of BOS, increasing sensitivity. By strictly matching patients with BOS and non-BOS patients, we aimed to decrease bias with respect to sex, age, use of immunosuppression, and disease before LTx. However, this study does have some limitations. The main limitation of this study is that the current results, based on this retrospective study, do not establish a causal link between the markers found in the serum and the histological features characterizing BOS. For the proteomics analysis, the FDR was set at 0.1 for protein and feature level allowing for identification of new proteins in this cohort. Candidate proteins such as PZP and ITIH3 need to be validated in a larger cohort. Unfortunately, no lung tissue was available from the patients included. Possibly, histological differences could explain why serum analysis of collagen neoepitopes was not significant. Even though all patients with BOS did have end-stage BOS, histologically, there could be differences in degree of inflammation, aberrant repair, and fibrosis within the airways. This complicates the interpretation of our findings as well as correlating the findings to the pathological processes in BOS lungs. Additional research, focusing on these markers in lung tissue is needed to further elucidate the role of these markers in BOS. The total number of patients included in this study is small due to the strict matching of patients with BOS and non-BOS patients, which decreases the power of this study. This could also be the reason that results for C1M were not significantly different between BOS and non-BOS. Also, not all patients had serum samples available for all timepoints, because of the retrospective nature of this study, which could influence results. Furthermore, it is not known if other diseases of patients have influenced the results in this study, since measurements in serum are not restricted to diseases in the lung and patients were not matched for comorbidities. Different dosages of immunosuppression could also have influenced results, even though patients were matched for type of immunosuppression used. Ideally, we would have used BAL fluid to validate our results. Hypothetically, BAL fluid can reflect pulmonary tissue concentrations of serum markers more accurately and is less affected by systemic processes, However, we had no availability to BAL fluid from these patients. Therefore, future validation of results is necessary in different media such as BAL fluid or lung tissue. Also, the staging for BOS severity changed in 2019, now also including stage 4 (FEV1 < 35% of baseline)^5^; however, due to the historical nature of our data with inclusion until 2017, we decided to apply the earlier staging criteria of the ISHLT.^18^

## Conclusion

In conclusion, this study suggests that several pathways are involved in the pathology of BOS, reflecting a complex interplay between both aberrant repair and inflammation in BOS. The results should be further investigated in larger, prospective studies to expand the knowledge on their role in the development of BOS.

## Author contributions

Concept and design of this study were performed by EA van der Ploeg, A Faiz, JK Burgess, BN Melgert, DJ Leeming, JMB Sand, and CT Gan. Acquisition of the data was performed by G Teitsma, A Faiz, A Sanchez Brotons, N Govorukhina, DJ Leeming, JMB Sand, and P Horvatovich. Interpretation of the data was performed by EA van der Ploeg, A Faiz, CT Gan, and JK Burgess. Drafting of the manuscript was done by EA van der Ploeg and A Faiz. G Teitsma, P Horvatovich, JK Burgess, A Sanchez Brotons, N. Govorukhina, JMB Sand, DJ Leeming, BN Melgert, and CT Gan critically revised the manuscript.

## Disclosure statement

The authors declare the following financial interests/personal relationships which may be considered as potential competing interests: CT Gan reports financial support was provided by Dutch Foundation for Asthma Prevention. JK Burgess reports financial support was provided by Nederlandse Organisatie voor Wetenschappelijk Onderzoek Utrecht. DJ Leeming and JMB Sand report a relationship with Nordic Bioscience that includes employment and equity or stocks. CT Gan reports a relationship with Chiesi Pharmaceuticals Inc. that includes consulting or advisory and speaking and lecture fees. The other authors declare that they have no known competing financial interests or personal relationships that could have appeared to influence the work reported in this paper.

This research was supported by a grant from 10.13039/501100004344Stichting Astma Bestrijding, grant number 2019/011 to C.T. Gan and 10.13039/501100003246Nederlandse Organisatie voor Wetenschappelijk Onderzoek (NOW) Aspasia premie subsidienummer 015.013.010 to J.K. Burgess.

## Data Availability

All data generated or analyzed during this study are included in this article and its supplementary material files. Further enquiries can be directed to the corresponding author.

## Footnotes

Appendix A Supplementary data associated with this article can be found in the online version at doi:10.1016/j.jhlto.2025.100303.
